# Practical sensorless aberration estimation for 3D microscopy with deep learning

**DOI:** 10.1364/OE.401933

**Published:** 2020-09-15

**Authors:** Debayan Saha, Uwe Schmidt, Qinrong Zhang, Aurelien Barbotin, Qi Hu, Na Ji, Martin J. Booth, Martin Weigert, Eugene W. Myers

**Affiliations:** 1 Max Planck Institute of Molecular Cell Biology and Genetics, Dresden, Saxony 01307, Germany; 2 Center for Systems Biology Dresden, Dresden, Saxony 01307, Germany; 3 University of California, Berkeley, California 94720, USA; 4 University of Oxford, Department of Engineering Science, Oxford OX13PJ, UK; 5 Institute of Bioengineering, School of Life Sciences, EPFL, Lausanne CH1015, Switzerland; 6 martin.booth@eng.ox.ac.uk; 7 martin.weigert@epfl.ch; 8 myers@mpi-cbg.de

## Abstract

Estimation of optical aberrations from volumetric intensity images is a key step in sensorless adaptive optics for 3D microscopy. Recent approaches based on deep learning promise accurate results at fast processing speeds. However, collecting ground truth microscopy data for training the network is typically very difficult or even impossible thereby limiting this approach in practice. Here, we demonstrate that neural networks trained only on simulated data yield accurate predictions for real experimental images. We validate our approach on simulated and experimental datasets acquired with two different microscopy modalities and also compare the results to non-learned methods. Additionally, we study the predictability of individual aberrations with respect to their data requirements and find that the symmetry of the wavefront plays a crucial role. Finally, we make our implementation freely available as open source software in Python.

## 1. Introduction

Image quality in volumetric microscopy of biological samples is often severely limited by optical aberrations due to refractive index inhomogeneities inside the specimen [1,2]. Adaptive optics (AO) is widely used to correct for these distortions via optical elements like deformable mirrors or spatial light modulators [3,4]. Successful implementation of AO requires aberration measurements at multiple locations within the imaging volume [5]. This can be achieved by creating point sources such as embedded fluorescent beads [6] or optically induced guide stars [7], and then sensing the wavefront either *directly* via dedicated hardware (e.g. Shack-Hartman wavefront sensors [8,9]) or *indirectly* from the intensity image of the point source (PSF) alone [10,11]. Due to its special hardware requirements, and its reliance on a point-scanning configuration, direct wavefront sensing can be cumbersome to implement and too slow for volumetric imaging of living samples [12]. In contrast, indirect wavefront sensing - or *phase retrieval* - offers the possibility to infer the aberration at multiple locations, across the entire volume simultaneously, without additional optical hardware [13,14]. Establishing a fast and accurate phase retrieval method from intensity images of point sources is therefore an important step for making AO more accessible to live imaging of large biological samples.

Classical approaches to phase retrieval include alternating projection methods such as Gerchberg-Saxton (GS) [11,15] or parameterized PSF fitting methods such as ZOLA [16] or VIPR [17]. While projection methods are typically fast but can perform poorly especially for noisy images, PSF fitting methods can achieve excellent results yet are relatively slow. Over the last years, deep learning-based approaches using convolutional neural networks (CNNs) have proven to be powerful and computationally efficient for image-based classification and regression tasks for microscopy images [18,19]. Recently, several studies demonstrated that deep learning-based phase retrieval can produce accurate results at fast processing speeds [20–25], however they fall short regarding their practical applicability. Some of these approaches [22–24] used purely simulated synthetic data, where generalizability to real microscopy images is unclear. Others focused on specific microscopy acquisition modes (such as using biplanar PSFs [20]) or microscopy setups that allow to collect large sets of experimental ground truth data for training and prediction [21,25], thus limiting this approach in practice. Moreover, most studies lack comparison against strong classical phase retrieval methods that are used in practice. As a result, the practical applicability of these approaches in experimental microscopy settings remains unclear.

In this paper we demonstrate for the first time that CNNs trained on appropriately generated synthetic data can be successfully applied to real images acquired with different microscopy modalities thereby avoiding the difficult or even impossible collection of experimental training data. Specifically, we generate synthetic 3D bead images with random aberrations via a realistic image formation model that matches the microscope setup, and we use a simple CNN architecture (which we call PHASENET) to directly predict these aberrations from the given volumetric images. We demonstrate the efficacy of our approach on two distinct microscopy modalities: i) a point-scanning microscope where single-mode aberrations were introduced in the illumination path, and ii) a widefield microscope where random-mode aberrations were introduced in the detection path. In contrast to other works [20,22], we also quantitively compare the speed and accuracy of PHASENET with the two popular state-of-the-art methods GS and ZOLA and find that PHASENET leads to competitive results yet is orders of magnitude faster. Finally, we demonstrate that the number of focal planes required for accurate prediction with PHASENET is related to different symmetry groups of the Zernike modes.

## 2. Method

**Fig. 1.**
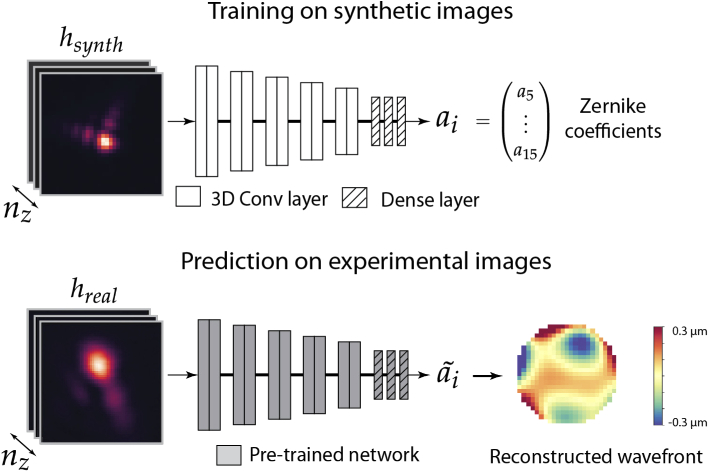
Overview of our approach: We train a CNN (PHASENET) with synthetic PSFs ${\text{h}}_{\text{synth}}$ (${\text{n}}_{\text{z}}$ axial planes) generated from randomly sampled amplitudes of Zernike modes ${\text{a}}_{\text{i}}\;$. The trained network is then used to predict the amplitudes $\tilde{{\text{a}}_{\text{i}}}$ from experimental bead images ${\text{h}}_{\text{real}}$. The predicted amplitudes $\tilde{{\text{a}}_{\text{i}}}$ are then used to reconstruct the wavefront.

Let h(x,y,z) be the acquired image of a bead (point spread function, PSF) and let $\varphi (k_{x},\;k_{y})$ be the *wavefront aberration*, i.e. the phase deviation from an ideal wavefront defined on the back pupil with coordinates $k_{x},\;k_{y}$. The wavefront aberration φ is then decomposed as a sum of Zernike polynomials/modes

(1) $$\varphi (k_{x},\;k_{y})=\sum_{i}a_{i}Z_{i}(k_{x},\;k_{y})$$

with $Z_{i}(k_{x},\;k_{y})$ being the i-th (Noll indexed) Zernike mode and $a_{i}$ the corresponding amplitude [26,27]. The problem of phase retrieval is then to infer these amplitudes $a_{i}$ from h(x,y,z). Our approach (PHASENET) uses a CNN model that takes a 3D image as an input and directly outputs the amplitudes $a_{i}$. Importantly, the model is trained on synthetically created data first and only then applied to real microscopy images (cf. Fig. 1). That way, we avoid the acquisition of experimental training images with precisely known aberrations, which often is difficult or outright impossible (*e.g.* for sensorless setups).

### 2.1 Synthetic training data

To generate training data for a specific microscope setup, we synthetically create pairs ${\left(a_{i}^{n},h_{synth}^{n}\right)}_{n\in N}$ of randomly sampled amplitudes $a_{i}^{n}$ and corresponding 3D PSFs $h_{synth}^{n}$. We use only the first 11 non-trivial Zernike modes $a_{i}^{n}=\;(a_{5}^{n},\;\cdots ,\;a_{15}^{n})$, excluding *piston*, *tip*, *tilt* and *defocus*, and generate randomly aberrated PSFs by uniformly sampling $a_{i}^{n}\in [-0.075\mu m,\;0.075\mu m]$ corresponding to the experimentally expected amplitude range. Given a wavefront $\varphi^{n}(k_{x},\;k_{y})=\sum_{i}a_{i}^{n}Z_{i}$, we compute the corresponding intensity image as:

(2) $${\text{h}}_{\text{synth}}^{\text{n}}(\text{x, y, z})\text{ = }{\left|ℑ\;\left[\text{P}({\text{k}}_{\text{x}}\text{,}{\text{k}}_{\text{y}})\;e^{2\pi i\varphi^{n}(k_{x},\;k_{y})/\lambda }\;e^{-2\pi iz\sqrt{\frac{n_{0}^{2}}{\lambda^{2}}-k_{x}^{2}-k_{y}^{2}}}\right]\right|}^{2}$$

where ℑ[·] is the 2D Fourier transform with respect to the pupil coordinates $k_{x}\;$ and $k_{y}$, *λ* is the wavelength, $n_{0}$ is the refractive index of the immersion medium, $\varphi^{n}(k_{x},\;k_{y})=\;\sum_{i=5}^{15}a_{i}^{n}Z_{i}(k_{x},\;k_{y})$ is the wavefront aberration, and $P(k_{x},\;k_{y})$ is the amplitude of the pupil function [28]. Since we do not consider amplitude attenuation, we simply set $P(k_{x},\;k_{y})\;=\;1_{k_{x}^{2}+k_{y}^{2}<{\left(\frac{NA}{\lambda }\right)}^{2}}$ with NA being the numerical aperture of the objective. To accommodate for a finite bead size, we then convolve ${\text{h}}_{\text{synth}}^{\text{n}}$ with a sphere of appropriate diameter (depending on the experiment) and add realistic Gaussian and Poisson noise.

### 2.2 PHASENET

The CNN architecture (PHASENET) is shown in Fig. 1 and consists of five stacked blocks, each comprising two 3×3×3 convolutional layers (with stride 1 and the number of channels doubling every block starting with 8) and one max-pooling layer (only along the lateral dimensions), followed by two dense layers (64 channels) and a final dense layer having the same number of neurons as the number of Zernike amplitudes to be predicted (11 in our case). We use *tanh* as activation function for all layers except the last, where we use linear activation. This results in a rather compact CNN model with a total of 0.9 million parameters which we found to perform equally well for our task as more complex architectures (e.g. ResNet [22], cf. Fig. S9 in Supplement 1). The 3D input size of PHASENET (e.g. 32×32×32) is fixed for each experimental setting. We simulate 3D PSFs ${\text{h}}_{\text{synth}}^{\text{n}}$ and the corresponding amplitudes $a_{i}^{n}$ which form the input and output of the network, respectively (cf. Fig. 1). To prevent overfitting, we use a data generator to continuously create random batches of training data pairs during the training process. We minimize the mean squared error (MSE) between predicted and ground truth (GT) amplitudes and train each model for 50000 steps and batch size 2 on a GPU (NVIDIA Titan Xp) using the Adam optimizer [29] with learning rate $1\;\cdot \;10^{-4}$ for a total training time of 24 h. Our synthetic training generation pipeline as well as the PHASENET implementation based on Keras [30] can be found at https://github.com/mpicbg-csbd/phasenet.

### 2.3 Experimental data

We use two different microscope setups (Point Scanning and Widefield) to demonstrate the applicability of this technique on real microscopy data.

#### 2.3.1 Point scanning

This is a point-scanning microscope designed for STED microscopy, equipped with a 1.4NA oil immersion ($n_{0}\;=1.518$) objective and a λ=755nm illumination laser (cf. Fig. S1(a) in Supplement 1 and described in [31]). For these experiments, the system was operated without the STED function activated – in effect as a point scanning confocal microscope with open pinhole. Single Zernike mode aberrations for $Z_{5}$ (oblique astigmatism) to $Z_{15}$ (oblique quadrafoil) within an amplitude range of ±0.11μm were introduced in the illumination path via a spatial light modulator (SLM). The backscattering signal of 80nm gold beads was then measured using a photomultiplier tube and the stage axially and laterally shifted resulting in n=198 aberrated 3D bead images of size 32×32×32 with isotropic voxel size 30nm. We generated synthetic training data using the given microscope parameters and random amplitudes$\;(a_{5},\cdots ,\;a_{15})$ in the range of ±0.075μm (cf. Section 2.1). We then trained a PHASENET model as explained in Section 2.2.

#### 2.3.2 Widefield

This is a custom-built epifluorescence microscope with a 1.1NA water immersion objective and a λ=488nm illumination laser (cf. Fig. S1(b) in Supplement 1). Mixed Zernike mode aberrations comprising $Z_{5}\;-\;Z_{10}$ (lower order) or $Z_{5}\;-\;Z_{15}$ (higher order) were introduced in the detection path via a deformable mirror (DM). We used an amplitude range of ±0.075μm for each mode. The images of 200nm fluorescent beads were recorded at different focal positions, resulting in n=100 aberrated 3D bead images of size 50×50×50 with a voxel size of 86 nm laterally and 100nm axially. As before, we generated similar synthetic training data using the respective microscope parameters and trained a PHASENET model.

### 2.4. Evaluation and comparison with classical methods

We compare PHASENET against two classical iterative methods, GS (Gerchberg-Saxton, code from [11]) and ZOLA [16]. GS is an alternating projection method that directly estimates the wavefront aberration φ. ZOLA fits a realistic PSF model to the given image and returns the present Zernike amplitudes (Supp. Notes A). For both GS and ZOLA, we used 30 iterations per image, ZOLA additionally leveraging GPU-acceleration (NVIDIA Titan Xp). For every method we quantify the prediction error by first reconstructing the wavefront from the predicted Zernike amplitudes (for PHASENET and ZOLA) and then computing the root mean squared error (RMSE, in µm) of the difference between the predicted and the ground truth wavefront.

## 3. Results

### 3.1 Point scanning

**Fig. 2.**
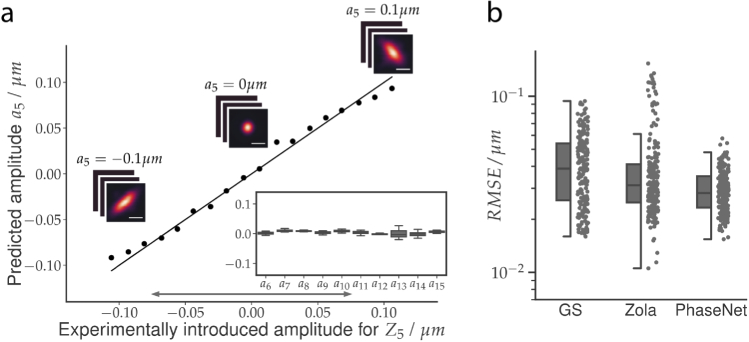
Measurement of single Zernike mode aberrations for Point Scanning data: a) PHASENET predictions on images with experimentally introduced oblique astigmatism ${\text{Z}}_{5}$ (see Fig. S2 in Supplement 1 for modes ${\text{Z}}_{6}\;-\;{\text{Z}}_{15}$). Shown are ground truth vs. the predicted amplitude ${\text{a}}_{5}$ (black dots), perfect prediction (solid black line), and the upper/lower bounds of amplitudes used during training (gray arrow). The inset shows the distribution of predicted non-introduced modes $\left({\text{a}}_{6},\;\cdots ,\;{\text{a}}_{15}\right)$. Scalebar 500 nm. b) RMSE for PHASENET and compared methods (GS and ZOLA) on all images. Boxes show interquartile range (IQR), lines signify median, and whiskers extend to 1.5 IQR.

We first investigated the performance of PHASENET on the data from Point Scanning microscope with experimentally introduced single-mode aberrations (cf. Fig. 2). This gives us the opportunity to assess the performance of all methods for each Zernike mode and amplitude in isolation. Here, the respective PHASENET model trained on synthetic PSFs achieved good wavefront reconstruction with the predicted and ground truth wavefront having a median RMSE of 0.025 µm (compared to the RMSE 0.15 µm of the input wavefronts), thus validating our approach (cf. Fig. S2 in Supplement 1). We then applied the model on the experimental images, yielding amplitude predictions $\left(a_{5},\;\cdots ,\;a_{15}\right)$ for each 3D input. In Fig. 2(a) we show the results for $Z_{5}$ (oblique astigmatism). As can be seen, the predicted amplitude $a_{5}$ exhibits good agreement with the experimental ground truth, even outside the amplitude range used for training (indicated by the gray arrow). Importantly, the predicted amplitudes for the non-introduced modes $\left(a_{6},\;\cdots ,\;a_{15}\right)$ were substantially smaller, indicating only minor cross-prediction between modes (cf. inset in Fig. 2(a)). The same can be observed for all other modes $Z_{6}\;-\;Z_{15}$ (cf. Fig. S3 and Fig. S4 in Supplement 1 for reconstructed wavefronts).

We next quantitatively compared the results of PHASENET with predictions obtained with GS and ZOLA. Here, PHASENET achieves a median RMSE between predicted and ground truth wavefronts of 0.028 µm across all acquired images (n = 198), which is comparable to the prediction error on synthetic PSFs. At the same time GS (0.039 µm) and ZOLA (0.031 µm) performed slightly worse (cf. Fig. 2(b), Fig. S8 in Supplement 1). This demonstrates that a PHASENET model trained only on synthetic images can indeed generalize to experimental data and achieve better performance than classical methods. Interestingly, although this dataset uses a high numerical aperture objective, PHASENET achieves high accuracy despite using only a scalar PSF model (2) which neglects vectorial effects in the PSF simulation [17]. Crucially, predictions with PHASENET were obtained orders of magnitude faster than with both GS and ZOLA (cf. Table 1). Whereas it took only 4 ms for PHASENET to process a single image, it required 0.12s for GS and 17.1s for ZOLA. The speed advantage of PHASENET is even more pronounced when predicting batches of several images simultaneously (cf. Table 1).

**Table 1.** Runtime of all methods for aberration estimation from a single (n = 1) and multiple (n = 50) PSFs of size 32×32×32.

Method	single (n = 1)	batched (n = 50)
GS	0.120 s	6.2 s
ZOLA	17.1 s	838 s
PHASENET	0.004 s	0.033 s

### 3.2 Widefield

**Fig. 3.**
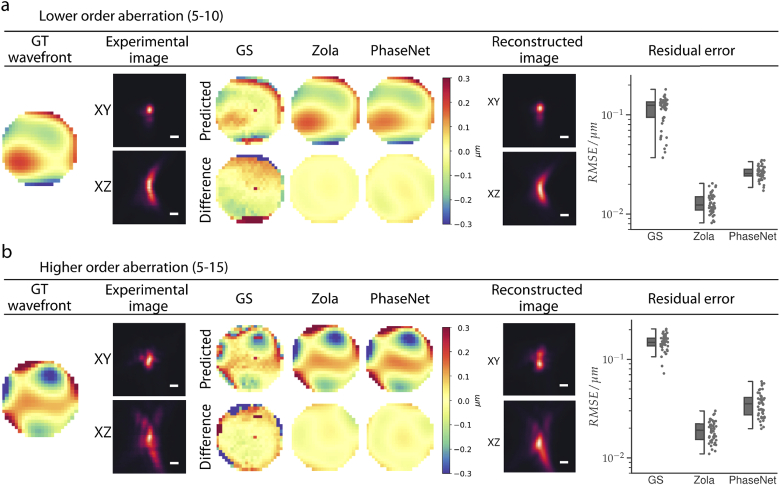
Results for Widefield data with mixed-modes aberrations: a) Predictions for lower order modes $({\text{Z}}_{5}\;-\;{\text{Z}}_{10}):$ We show the ground truth (GT) wavefront, lateral (XY) and axial (XZ) midplanes of the experimental 3D image, the reconstructed wavefront and their GT difference for all methods (Gerchberg-Saxton/GS [11], ZOLA, PHASENET), and the reconstructed image from the PHASENET prediction. We further depict the RMSE for all n = 50 experimental PSFs. Boxes show interquartile range (IQR), lines signify median, and whiskers extend to 1.5 IQR. b) Same results but including higher order modes ${\text{Z}}_{5}\;-\;{\text{Z}}_{15}$. Scalebar: 500 nm.

We next explored the applicability of our approach to the widefield microscope modality, where mixed-mode aberrations were randomly introduced. The PHASENET model trained on appropriate synthetic data achieved a median RMSE of 0.022 µm (compared to RMSE 0.14 µm of the input wavefronts) indicating again good wavefront reconstruction (Fig. S5 in Supplement 1). We then applied the trained model on the experimental bead images. In Fig. 3(a) we show results for PHASENET, GS, and ZOLA for images with introduced modes Z_5_ − Z_10_ (lower order). The reconstructed wavefronts for both PHASENET and ZOLA exhibit qualitatively good agreement with the ground truth, whereas GS noticeably underperforms (cf. Fig. S6 in Supplement 1). Similarly, the calculated RMSE across all images (n = 50) for GS (0.124 µm) is substantially larger than for PHASENET (0.025 µm) and ZOLA (0.012 µm). The same results can be observed when predicting images with higher order modes $Z_{5}\;-\;Z_{15}$ (Fig. 3(b)). As expected, RMSE values increased slightly compared to the lower order modes for all methods, with 0.148 µm for GS, 0.035 µm for PHASENET, and 0.019 µm for ZOLA (more examples can be found in Fig. S7 in Supplement 1). Although ZOLA yields slightly better RMSE than PHASENET for this dataset, PHASENET again vastly outperforms ZOLA and GS in terms of prediction time by being orders of magnitude faster (cf. Table S1 in Supplement 1).

### 3.3 Number of input planes

**Fig. 4.**
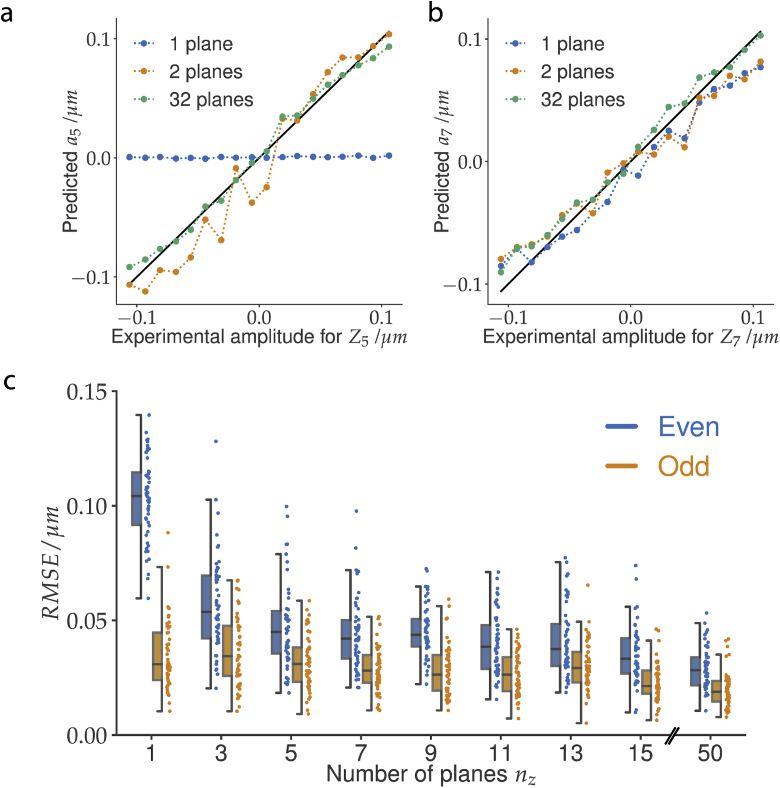
Results for varying number of input planes ${\text{n}}_{\text{z}}$: a) Ground truth vs. the predicted amplitude ${\text{a}}_{5}$ (oblique astigmatism) for single mode data Point Scanning and using PHASENET models with ${\text{n}}_{\text{z}}=\;1,\;2,\;32$. b) The same for ${\text{a}}_{7}$ (vertical coma). c) Prediction error (RMSE) on Widefield data (50 images) for PHASENET models trained with different ${\text{n}}_{\text{z}}$. We show the RMSE for odd (orange) and even (blue) Zernike modes separately. Boxes depict interquartile range (IQR), lines signify median, and whiskers extend to 1.5 IQR.

In both experiments so far, the 3D input of PHASENET consisted of many defocus planes ($n_{z}\;=\;32$ for Point Scanning and $n_{z}=50$ for Widefield). We set out to determine whether accurate aberration prediction is still possible with substantially fewer planes. We therefore trained several PHASENET models with varying $n_{z}$ and applied them to experimental images (cf. Supp. Notes B). In Figs. 4(a) and (b) we show predictions with $n_{z}\in \{1,\;2,\;32\}$ for single-mode aberrations $Z_{5}$ (oblique astigmatism) and $Z_{7}$ (vertical coma). Interestingly, we find that in the case of Z_5_ at least n_z_ ≥ 2 planes are needed for meaningful predictions, whereas in the case of Z_7_ already a single plane $\left(n_{z}=1\right)$ yields satisfactory results. This can be explained by observing that for purely $Z_{5}$ aberrations (i.e. $a_{i\neq 5}=\;0$), flipping the sign of the aberration amplitude $a_{5}^{\prime }=\;-a_{5}$ leads to a 3D PSF that is mirrored along the optical axis. Predicting the amplitude $a_{5}$ from a single image plane is therefore inherently ambiguous. To further examine this, we grouped the Zernike modes into the classes even and odd depending on the symmetry of the wavefront $\left(even:\;Z_{5},\;Z_{6},\;Z_{11},\;...,\;odd:\;Z_{7},\;Z_{8},\;Z_{9},\;...\right)$ and calculated the prediction for each class separately. As expected, the RMSE decreases with increasing $n_{z}$ (Fig. 4(c)) for both classes. However, for even Zernike modes the prediction error is significantly higher than for odd modes, especially when using only few planes, in line with our earlier observation.

## 4. Conclusion

We demonstrated for the first time that deep learning-based phase retrieval with our proposed PHASENET model using only synthetically generated training data does generalize to experimental data from different microscopy setups and allows for accurate and efficient aberration estimation from experimental 3D bead images. On datasets from two different microscopy modalities we showed that PHASENET yields better (Point scanning dataset) or almost comparable (Widefield dataset) results than classical methods, while being orders of magnitude faster. This opens up the interesting possibility of using PHASENET to perform aberration estimation from multiple beads or guide stars across an entire volumetric image in a real-time setting on the microscope during acquisition. We further investigated how prediction quality depends on the number of defocus planes ${\text{n}}_{\text{z}}$ and found that odd Zernike modes are substantially easier to predict than even modes for the same ${\text{n}}_{\text{z}}$.

Still, our approach may not be applicable to cases where the synthetic PSF model is inadequate for the microscope setup or where experimental data is vastly different from the data seen during training (a limitation that applies to most machine learning-based methods). In particular, the range of aberration amplitudes used in the synthetic generator should cover the range of experimentally expected aberrations. Moreover, for discontinuous wavefronts (such as double helix PSFs [32] or helical phase ramps [33]) the low-order Zernike mode representation is likely to be inadequate and PHASENET performance is therefore sub-optimal. Furthermore, our experimental data so far included only Zernike modes $Z_{n}\leq 15$, leaving the question open whether our approach would behave similarly for larger Zernike modes. Additionally, more advanced network architectures that explicitly leverage the physical PSF model might improve prediction accuracy. We believe that in the future our method can serve as an integral computational component of practical adaptive optics systems for microscopy of large biological samples.

## Notes

See Supplement 1 for supporting content.
