# Supplementary material for: Practical sensorless aberration estimation for 3D microscopy with deep learning
Source: Opt Express. 2020 Sep 15;28(20):29044–53. doi: 10.1364/OE.401933 (PMC7679184; doi:10.1364/OE.401933)
Supplement: Supplementary file 1 [file oe-28-20-29044-s001.pdf]

Supplemental Document

Optics EXPRESS

Practical sensorless aberration estimation for 3D microscopy with deep learning: supplement

DEBAYAN SAHA,[1,2] UWE SCHMIDT,[1,2] QINRONG ZHANG,[3] AURELIEN BARBOTIN,[4] QI HU,[4] NA JI,[3] MARTIN J. BOOTH,[4,6] MARTIN WEIGERT,[1,2,5,7] AND EUGENE W. MYERS[1,2,8]

[1] *Max Planck Institute of Molecular Cell Biology and Genetics, Dresden, Saxony 01307, Germany*
[2] *Center for Systems Biology Dresden, Dresden, Saxony 01307, Germany*
[3] *University of California, Berkeley, California 94720, USA*
[4] *University of Oxford, Department of Engineering Science, Oxford OX13PJ, UK*
[5] *Institute of Bioengineering, School of Life Sciences, EPFL, Lausanne CH1015, Switzerland*
[6] *martin.booth@eng.ox.ac.uk*
[7] *martin.weigert@epfl.ch*
[8] *myers@mpi-cbg.de*

This supplement published with The Optical Society on 15 September 2020 by The Authors under the terms of the Creative Commons Attribution 4.0 License in the format provided by the authors and unedited. Further distribution of this work must maintain attribution to the author(s) and the published article's title, journal citation, and DOI.

Supplement DOI: https://doi.org/10.6084/m9.figshare.12844652

Parent Article DOI: https://doi.org/10.1364/OE.401933

Practical sensorless aberration estimation for 3D microscopy with deep learning

Debayan Saha[1,2] , Uwe Schmidt[1,2] , Qinrong Zhang[3] , Aurelien Barbotin[4] , Qi Hu[4] , Na Ji[3] , Martin J. Booth[4,6] , Martin Weigert[1,2,5,7] and Eugene W. Myers[1,2,8]

[1] *Max Planck Institute of Molecular Cell Biology and Genetics, Dresden, Saxony 01307, Germany*
[2] *Center for Systems Biology Dresden, Dresden, Saxony 01307, Germany*
[3] *University of California, Berkeley, California 94720, USA*
[4] *University of Oxford, Department of Engineering Science, Oxford OX13PJ, UK*
[5] *Institute of Bioengineering, School of Life Sciences, EPFL, Lausanne, CH1015, Switzerland*
[6] *martin.booth@eng.ox.ac.uk*
[7] *martin.weigert@epfl.ch*
[8] *myers@mpi-cbg.de*

1. Supplementary notes

A. Classical Methods

Gerchberg-Saxton GS
We use an already published modified GS implementation (made available to us by the authors from [1]) and adapted it to the images from the experimental data sets (*e.g.* input image sizes). We checked the validity of the code by applying it to noise-free synthetic images, where the resulting wavefront was in good agreement to the ground-truth wavefront. Slightly better performance of GS was noticed on masking the periphery of the pupil plane, however the results are not shown here.

ZOLA
We used the ZOLA plugin (https://github.com/imodpasteur/ZOLA-3D) available for ImageJ/Fiji [2,3]. We wrote a macro in Fiji that loads the 3D image, automatically selects the pixel of maximal intensity, and calls the Zola plugin. The physical parameters of ZOLA were set according to the respective microscope setup. Good performance was obtained from the default camera parameters so they were not changed. We used 30 iteration steps for the optimization with GPU acceleration activated (NVIDIA Titan Xp). From the predicted amplitudes a_i we extracted the first 15 Zernike mode amplitudes and converted them to Noll order omitting a_1, a_2, a_3, a_4 (*piston*, *tip*, *tilt*, *defocus*).

B. Multi-plane experiments

Training data
We first generated isotropic 3D synthetic PSFs according to the microscope parameters with a fixed number of planes $n_{z_0} = 64$. From these we created smaller images with n_z planes by taking the $\pm 2k$-th plane from either side of the middle plane ($k = 0 \ldots n_x/2$).

Experimental data

From the 3D bead images acquired for single mode and random mode experiments (Point Scanning and Widefield), we took the maximum intensity plane as the mid plane and cropped the images as described above.

RMSE calculation for *odd*/even modes experiment

We reconstruct the wavefront for both PHASENET predictions and ground truth as a weighted sum of Zernike modes defined on the back pupil as described above. We then grouped both wavefronts into their even and odd components and computed the RMSE between predicted and ground truth component separately.

References

1. P. Kner, L. Winoto, D. A. Agard, and J. W. Sedat, “Closed loop adaptive optics for microscopy without a wave- front sensor,” in *Three-Dimensional and Multidimensional Microscopy: Image Acquisition and Processing XVII,* vol. 7570 (International Society for Optics and Photonics, 2010), p. 757006.
2. J. Schindelin, I. Arganda-Carreras, E. Frise, V. Kaynig, M. Longair, T. Pietzsch, S. Preibisch, C. Rueden, S. Saalfeld, B. Schmid *et al.*, “Fiji: an open-source platform for biological-image analysis,” Nat. methods **9**, 676–682 (2012).
3. C. A. Schneider, W. S. Rasband, and K. W. Eliceiri, “Nih image to imagej: 25 years of image analysis,” Nat. methods **9**, 671–675 (2012).
4. A. Barbotin, S. Galiani, I. Urbancˇicˇ, C. Eggeling, and M. J. Booth, “Adaptive optics allows sted-fcs measurements in the cytoplasm of living cells,” Opt. express **27**, 23378–23395 (2019).

2. Supplementary tables

Table S1. Runtime of all methods for aberration estimation from a single (n = 1) and multiple (n = 50) PSFs of size 50×50×50.

Method	single (n = 1)	batched (n = 50)
GS	0.98 s	49 s
ZOLA	28 s	1475 s
PHASENET	0.005 s	0.110 s

3. Supplementary figures

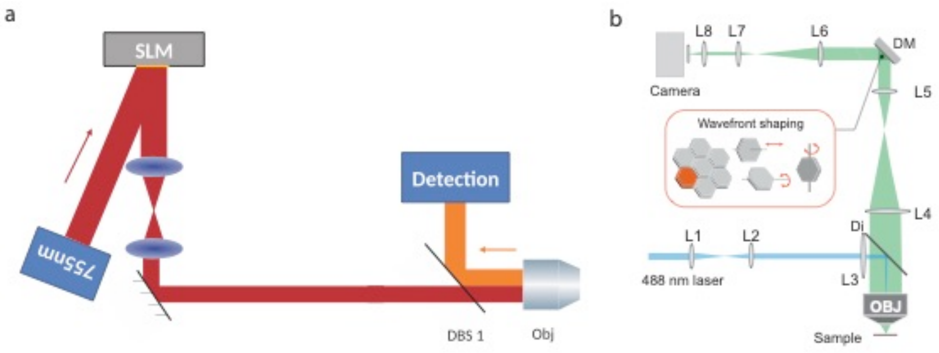

Fig. S1. Schematics of microscope set-ups. a) Adaptive point scanning microscope built around the modified, commercial RESOLFT microscope (Abberior Instruments, Germany). A 755*nm* laser (blue box with red beam path) was focused by a 100X/1.4 oil immersion objective (Obj; Olympus UPLSAPO). The phase of the laser was spatially modulated using a spatial light modulator (SLM; Hamamatsu LCOS X10468-02) which was relayed to the back focal plane of the objective using a pair of lenses (blue ellipses). The scattered light from a gold bead at focus of the objective was separated from the excitation light using a dichroic beam splitters (DBS 1) and was collected using a photomultiplier tube (blue box, Detection). (Figure reproduced with permission from [4], Creative Commons license CC BY 4.0). b) Home-built widefield fluorescence microscope. The output beam from a 488-nm continuous laser (blue beam path) was expanded to 8.4 mm in diameter before entering the microscope. And the beam was de-magnified to the sample plane by three lenses (L1,L2,L3) and a 25X/1.1 water immersion objective lens (OBJ; Nikon, CFI Apo LWD). A dichroic mirror (Di) was placed between L3 and the objective, reflecting illumination and transmitting emitted fluorescence. Emitted fluorescence (green beam path) was collected with the same objective, whose back focal plane was relayed to the deformable mirror (DM; Iris AO, PTT489) by a pair of lenses (L4-L5). The DM-reflected fluorescence was then focused and imaged on the camera by 3 lenses (L6-L7-L8).

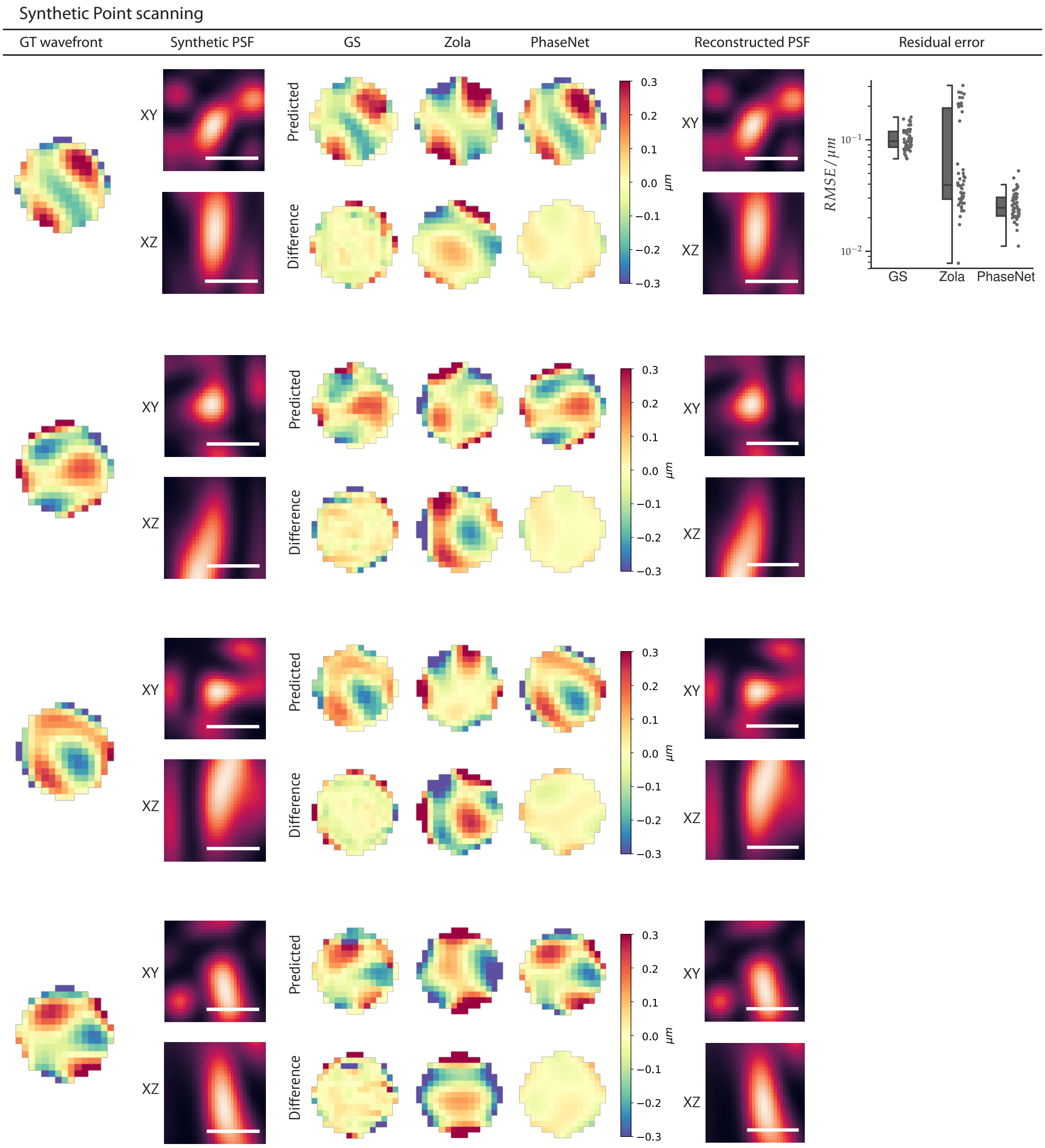

Fig. S2. Results on synthetic data. Random amplitudes of Zernike modes (Noll 5-15) in the range $[-0.075, 0.075]$ μm for each mode was used to create GT wavefronts. The corresponding 3D intensity PSFs were simulated, convolved with a sphere of $100nm$ diameter and noise was added to create 3D synthetic PSFs. We cropped the PSFs to an isotropic volume of 32 planes and (32 x 32) pixels to match the input shape of the network. Here we show examples of the ground truth wavefronts (reconstructed from the amplitudes of Zernike coefficients), lateral (XY) and axial (XZ) midplanes of the synthetic PSFs, the wavefronts estimated with Gerchberg-Saxton, ZOLA, and PHASENET (upper row) and their difference from the ground truth wavefronts (lower row), and the reconstructed PSFs from the PHASENET predictions. Additionally we show for all methods the error of the predicted wavefronts (root mean square error, RMSE) for 50 synthetic PSFs. Scalebar: $500nm$.

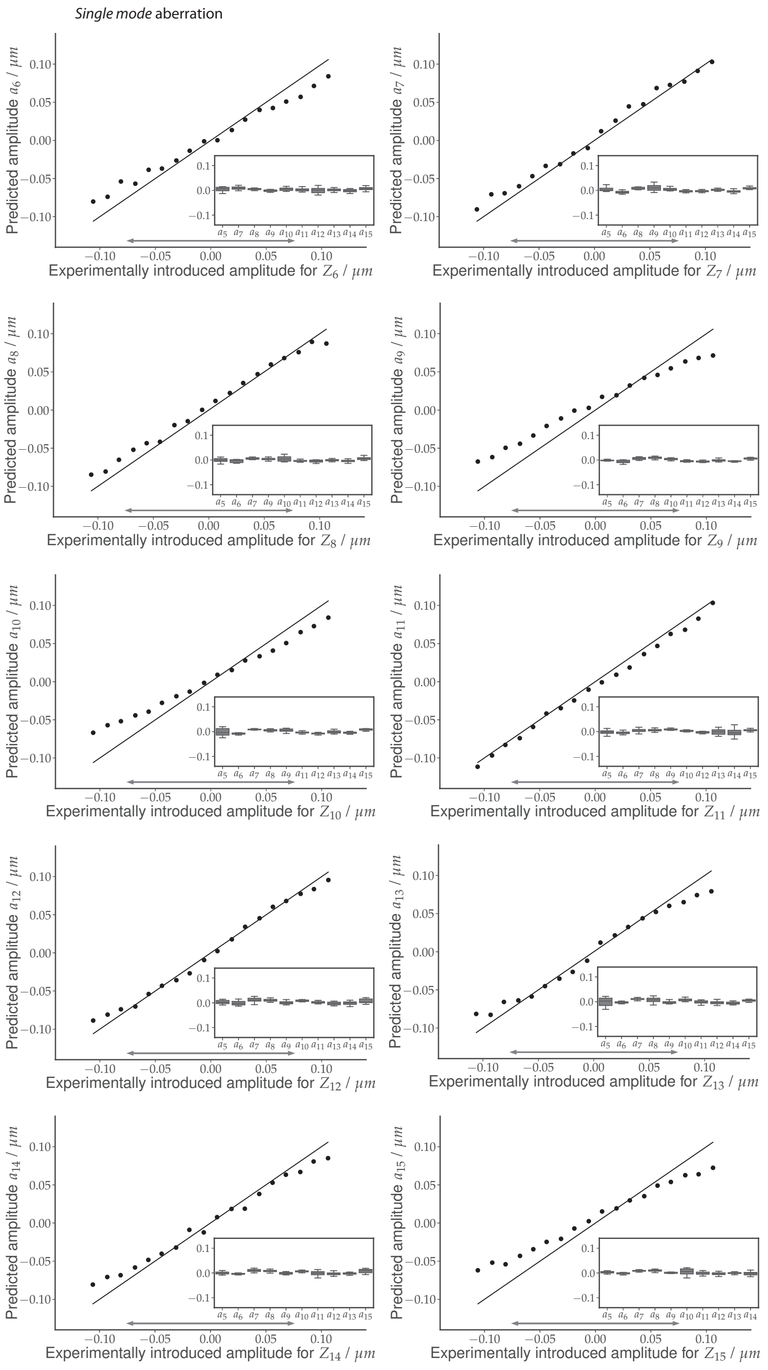

Fig. S3. Results of PHASENET on experimental *single mode* aberrations. A spatial light modulator was used to introduce single mode aberrations in the range $[-0.11, 0.11]\mu m$ and the respective 3D stacks ($n_z = 32\ planes$) of $80nm$ gold beads were acquired. A network trained on synthetic PSFs was used for predicting aberration amplitudes $(a_5, \ldots, a_{15})$ from the 3D stacks. Each graph shows the predicted amplitude for the single experimentally introduced Zernike mode *vs.* the ground truth amplitude of that mode for each single mode experiment. The inset of each graph depicts the distribution of predictions for the remaining non-introduced modes for that experiment. The solid black line of unit slope indicates perfect prediction, the gray arrow depict the upper and lower bound of amplitudes for which the network was trained ($a_i \in [-0.075, 0.075]\mu m$).

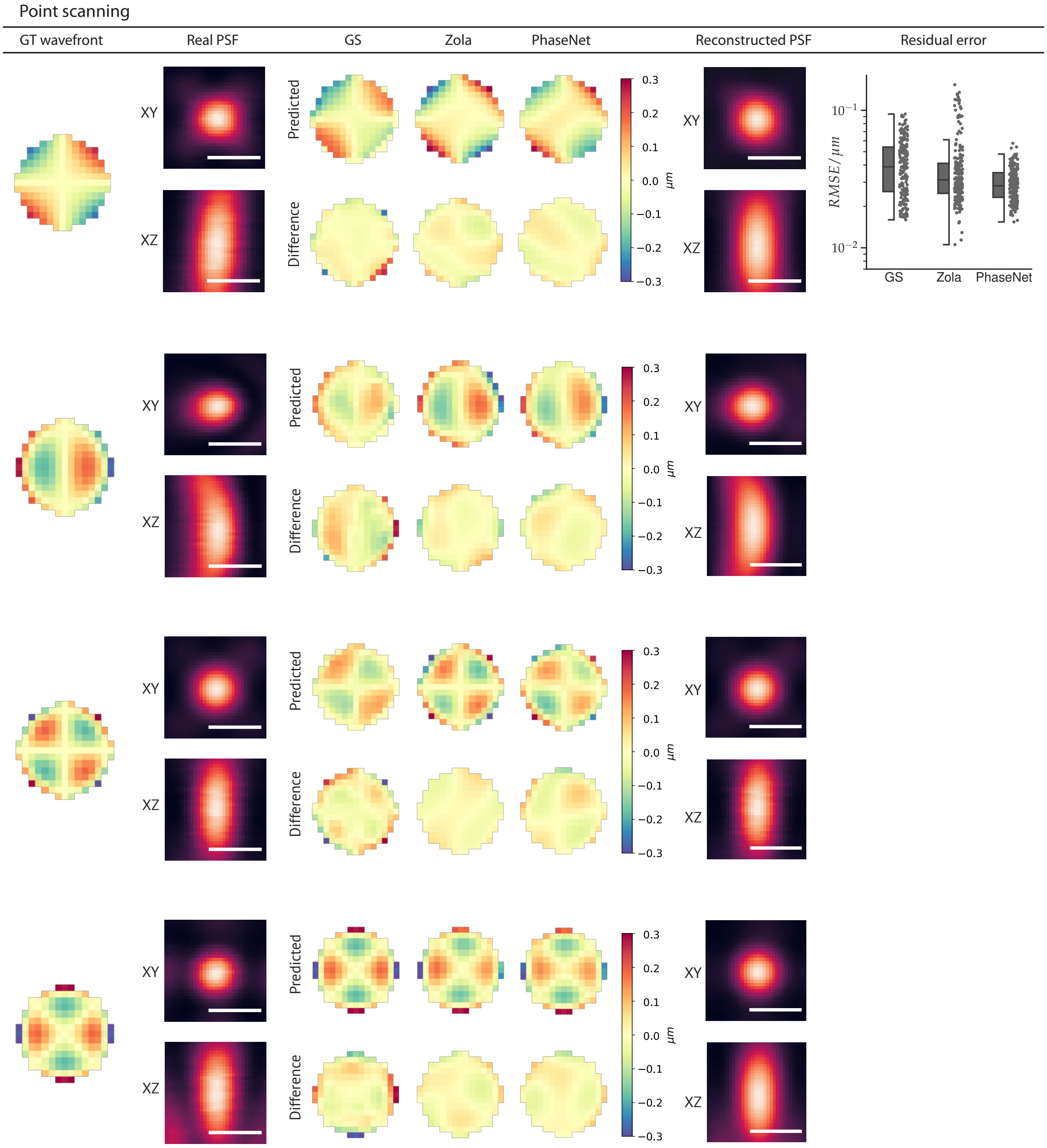

Fig. S4. Results of experimental *single mode* aberrations for Point Scanning microscope set-up. A spatial light modulator was used to introduce single mode aberrations in the range $[-0.11, 0.11]\mu m$ and the respective 3D stacks ($n_z = 32\, planes$) of 80 nm gold beads were acquired. A network trained on synthetic PSFs was used for predicting aberration amplitudes ($a_5, \ldots, a_{15}$) from the 3D stacks. Here we show examples of the ground truth wavefront (reconstructed from the amplitudes of Zernike coefficients), lateral (XY) and axial (XZ) midplanes of the synthetic PSF, the wavefront estimated with Gerchberg-Saxton, ZOLA, and PHASENET (upper row) and their difference from the ground truth wavefront (lower row), and the reconstructed PSF from the PHASENET prediction. Additionally we

show for all methods the error of the predicted wavefront (root mean square error, RMSE) for 198 PSFs. Scalebar: 500 *nm*.

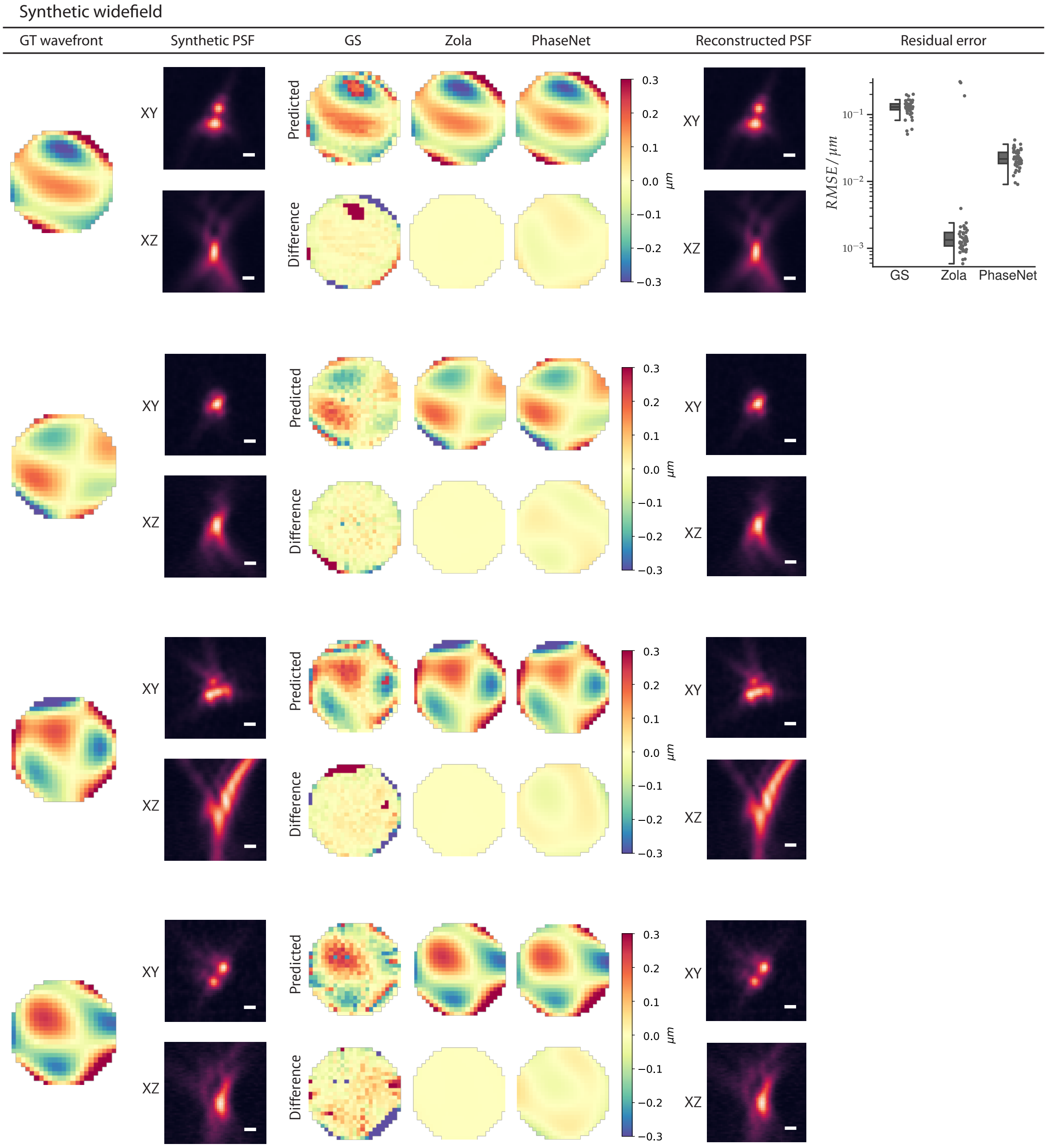

Fig. S5. Results on synthetic data. Random amplitudes of Zernike modes (Noll 5-15) in the range $[-0.075, 0.075]\mu m$ for each mode was used to create GT wavefronts. The corresponding 3D intensity PSFs were simulated, convolved with a sphere of $200nm$ diameter and noise was added to create a 3D synthetic PSFs. We cropped the PSFs to an isotropic volume of 50 planes and (50 x 50) pixels to match the input shape of the network. Here we show examples of the ground truth wavefronts (reconstructed from the amplitudes of Zernike coefficients), lateral (XY) and axial (XZ) midplanes of the synthetic PSFs, the wavefronts estimated with Gerchberg-Saxton, ZOLA, and PHASENET (upper row) and their difference from the ground truth wavefronts (lower row), and the reconstructed PSFs from the PHASENET predictions. Additionally we show for all methods the error of the predicted wavefronts (root mean square error, RMSE) for 50 synthetic PSFs. Scalebar: $500nm$.

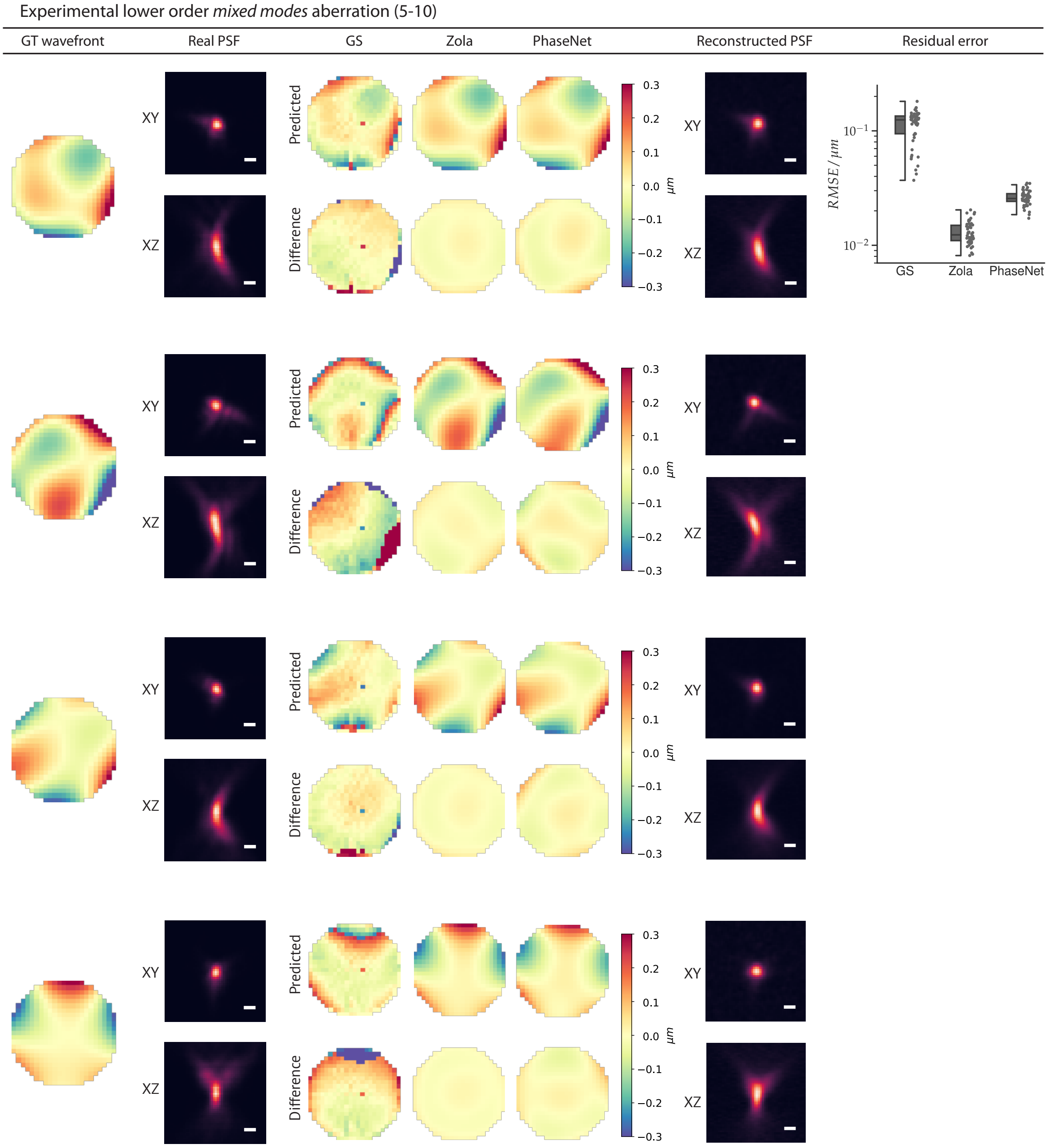

Fig. S6. Results of experimental low order *mixed modes* aberrations for widefield microscope set-up. A deformable mirror was used to introduce random amplitudes of low order modes (Noll 5-10) in the range $[-0.075, 0.075]\mu m$ for each mode. Here we show additional examples for: the ground truth wavefront (reconstructed from the amplitudes of Zernike coefficients), lateral (XY) and axial (XZ) midplanes of the acquired bead (PSF) stacks, the wavefront estimated with Gerchberg-Saxton, ZOLA, and PHASENET (upper row) and their difference from the ground truth wavefront (lower row), and the reconstructed PSF from the PHASENET prediction. Additionally we show for all methods the error of the predicted wavefront (root mean square error, RMSE) for 50 PSFs. Scalebar: 500 *nm*.

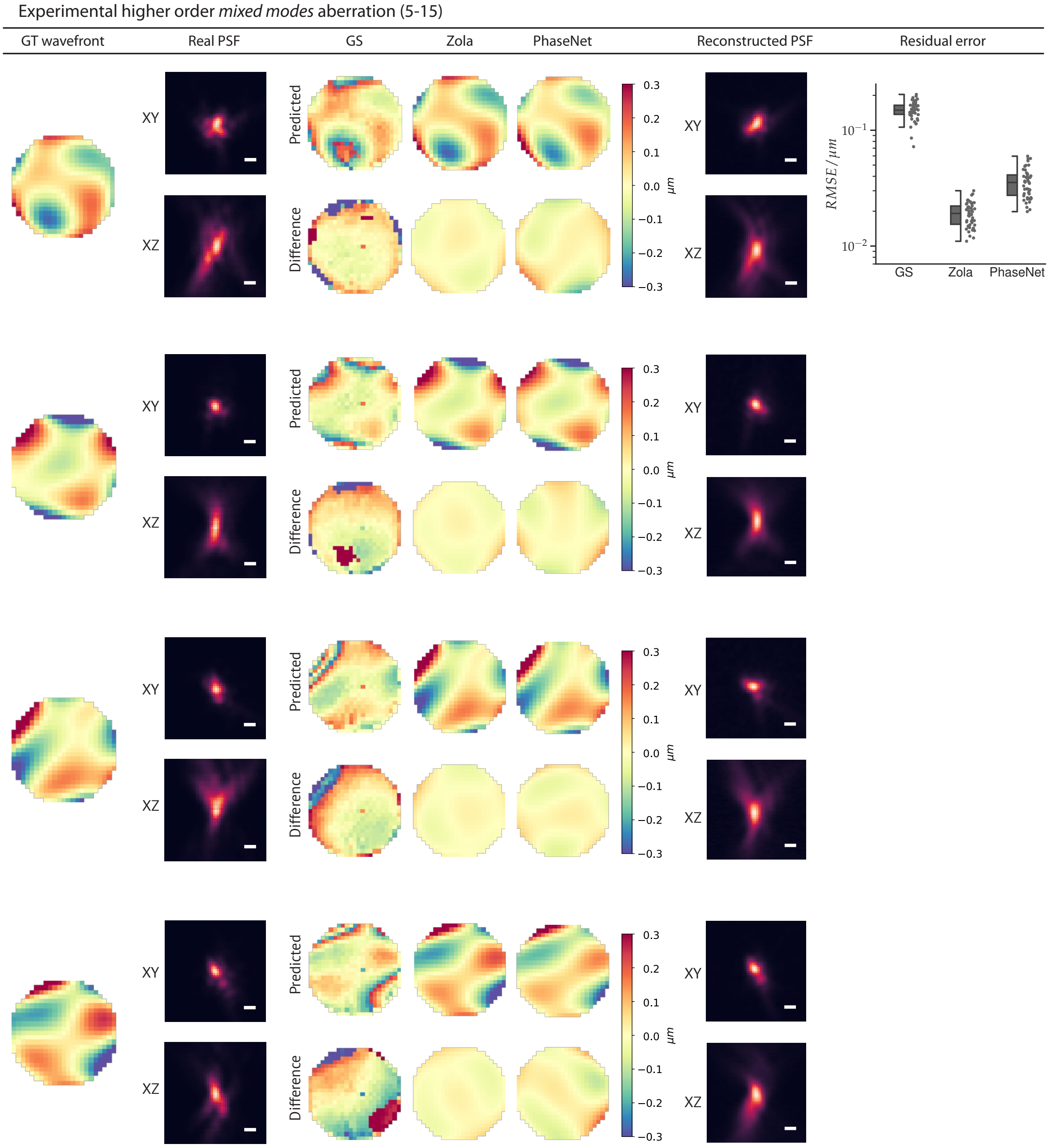

Fig. S7. Results of experimental high order *mixed modes* aberrations for widefield microscope set-up. A deformable mirror was used to introduce random amplitudes of low order modes (Noll 5-15) in the range $[-0.075, 0.075]\mu m$ for each mode. Here we show additional examples for: the ground truth wavefront (reconstructed from the amplitudes of Zernike coefficients), lateral (XY) and axial (XZ) midplanes of the acquired bead (PSF) stacks, the wavefront estimated with Gerchberg-Saxton, ZOLA, and PHASENET (upper row) and their difference from the ground truth wavefront (lower row), and the reconstructed PSF from the PHASENET prediction. Additionally we show for all methods the error of the predicted wavefront (root mean square error, RMSE) for 50 PSFs. Scalebar: 500 *nm*.

Zola Single mode aberration

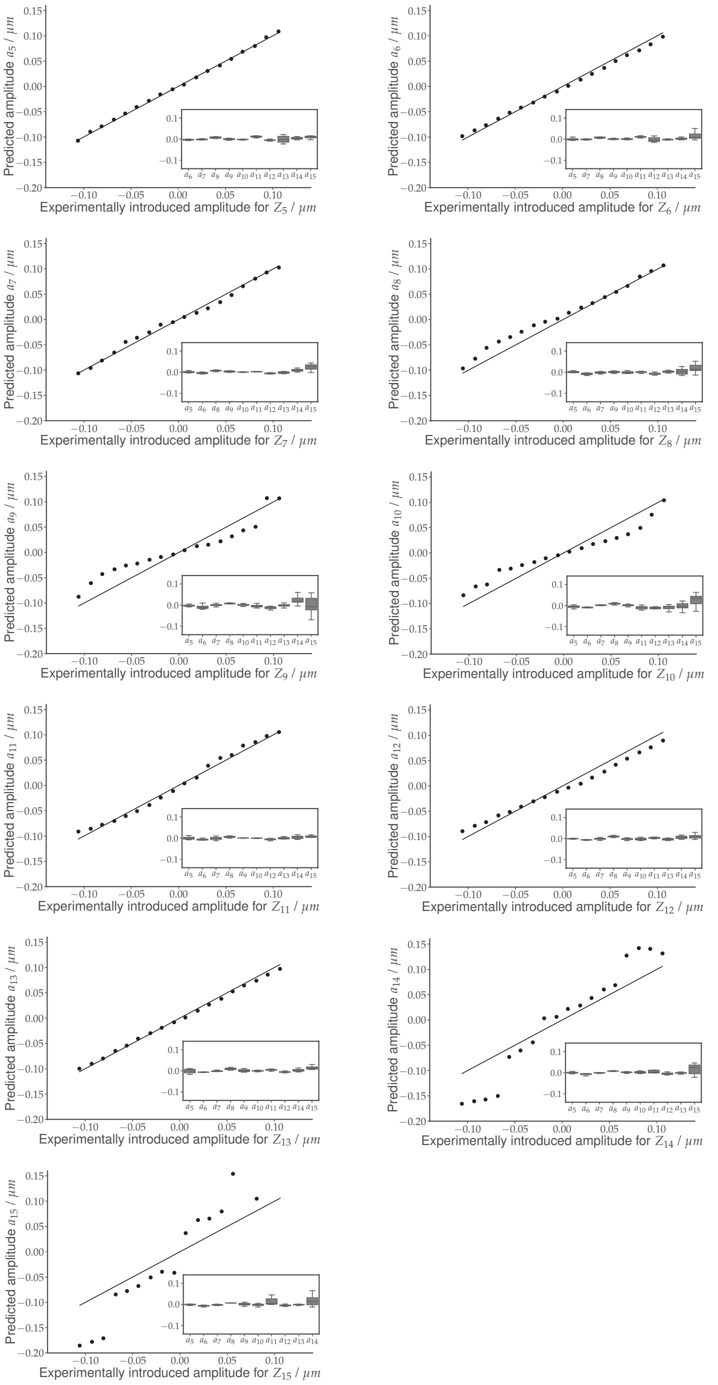

Fig. S8. Results of Zola on experimental *single mode* aberrations. A spatial light modulator was used to introduce single mode aberrations in the range $[-0.11, 0.11]\mu m$ and the respective 3D stacks ($n_z = 32\ planes$) of $80nm$ gold beads were acquired. Each graph shows the amplitude predicted by Zola for the single experimentally introduced Zernike mode *vs*. the ground truth amplitude of that mode for each single mode experiment. The inset of each graph depicts the distribution of predictions for the remaining non-introduced modes for that experiment. The solid black line of unit slope indicates perfect prediction.

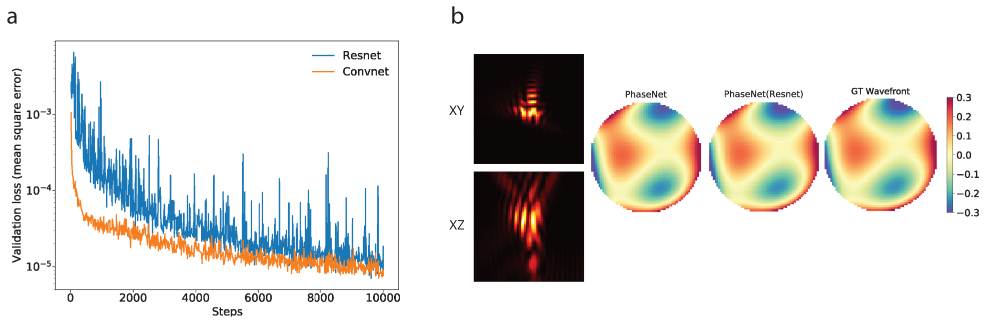

Fig. S9. Architecture comparison. We trained a regular PhaseNet model and a PhaseNet model with a ResNet architecture similar to [22] for 10000 parameter steps on synthetic noise free data (NA=1.0). a) The validation loss curves as a function of update steps. Note that the final loss is comparable for both models. b) Image of lateral/XY and axial/YZ mid slices of an example PSF along with PhaseNet and PhaseNet(Resnet) model prediction showing qualitatively almost identical wavefront estimations for both architectures.
